# Supplementary material for: Geographical Origin Differentiation of Rice by LC–MS-Based Non-Targeted Metabolomics
Source: Foods. 2022 Oct 23;11(21):3318. doi: 10.3390/foods11213318 (PMC9657058; doi:10.3390/foods11213318)
Supplement: Supplementary file 1 [file foods-11-03318-s001.zip › Tables S1-S3.pdf]

Table S1. Parameter settings of chromatographic conditions

Device name	Parameter conditions
Chromatographic column type	C18 column
Chromatographic column temperature	40 °C
Flow rate	0.2 mL min^{-1}
Mobile phase A	5 mmol/L ammonium acetate, pH 9.0
Mobile phase B	Methanol

Table S2. Chromatographic gradient program setting parameters

Time/min	A%	B%
0	98	2
1.5	98	2
3	0	100
10	0	100
10.1	98	2
11	98	2
12	98	2

Table S3. Condition parameter settings of MS

Device name	Parameter conditions
Scanning mode	Anion mode
Scanning range selection	m/z 100-1500
Spray voltage	33.5 kV;
Sheath gas velocity	35 L/min
Auxiliary gas flow rate	10 L/min
Ion transfer tube temperature	320 °C
Ion Import RF Level	60
Auxiliary gas heater temperature	350 °C
MS / MS Secondary Scanning	Data Dependence Scanning
