# Supplementary figures and images for: Geographical Origin Differentiation of Rice by LC–MS-Based Non-Targeted Metabolomics
Source: Foods. 2022 Oct 23;11(21):3318. doi: 10.3390/foods11213318 (PMC9657058; doi:10.3390/foods11213318)

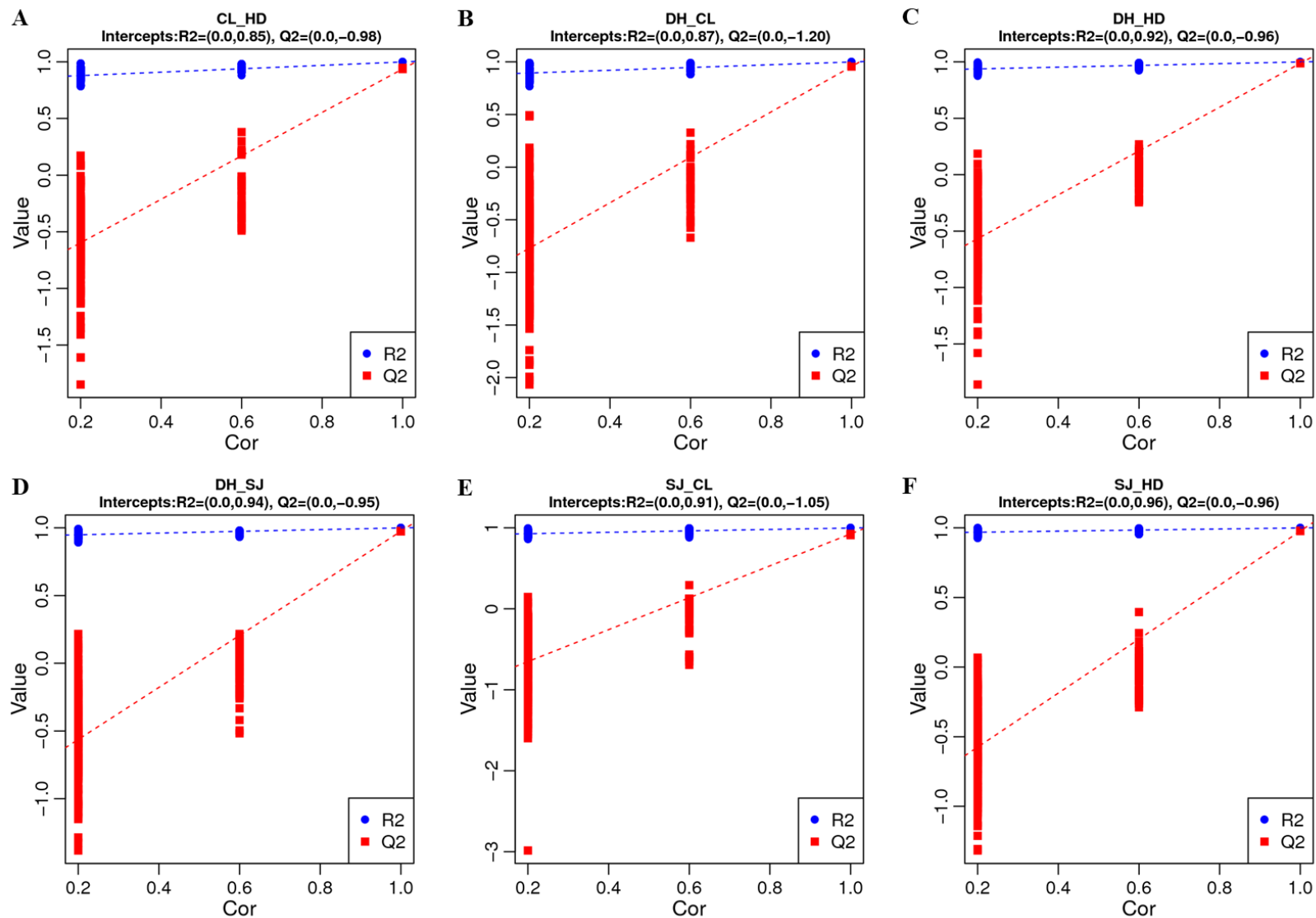

**Figure S1.** PLS-DA verification results. (A) CL vs HD; (B) CL vs DH; (C) DH vs HD; (D) DH vs SJ; (E) SJ vs CL; (F) SJ vs HD.

Supplement: Supplementary file 1 [file foods-11-03318-s001.zip › Figure S1.pdf]

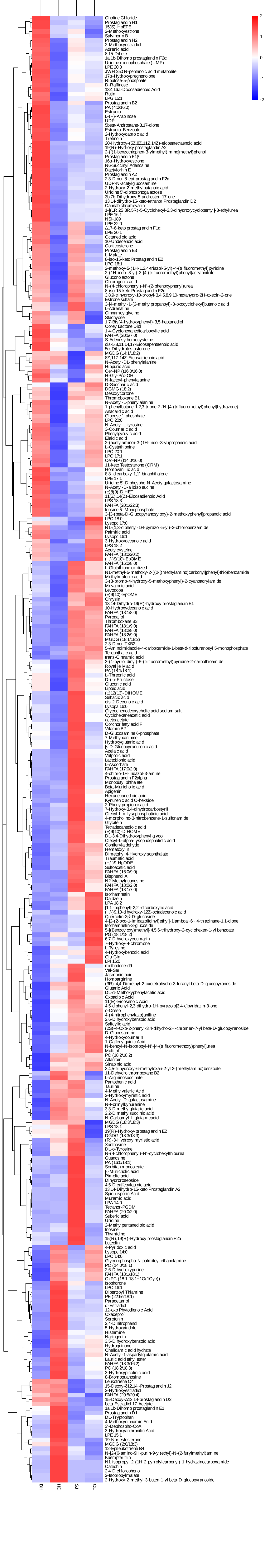

Supplement: Supplementary file 1 [file foods-11-03318-s001.zip › Figure S2.pdf]

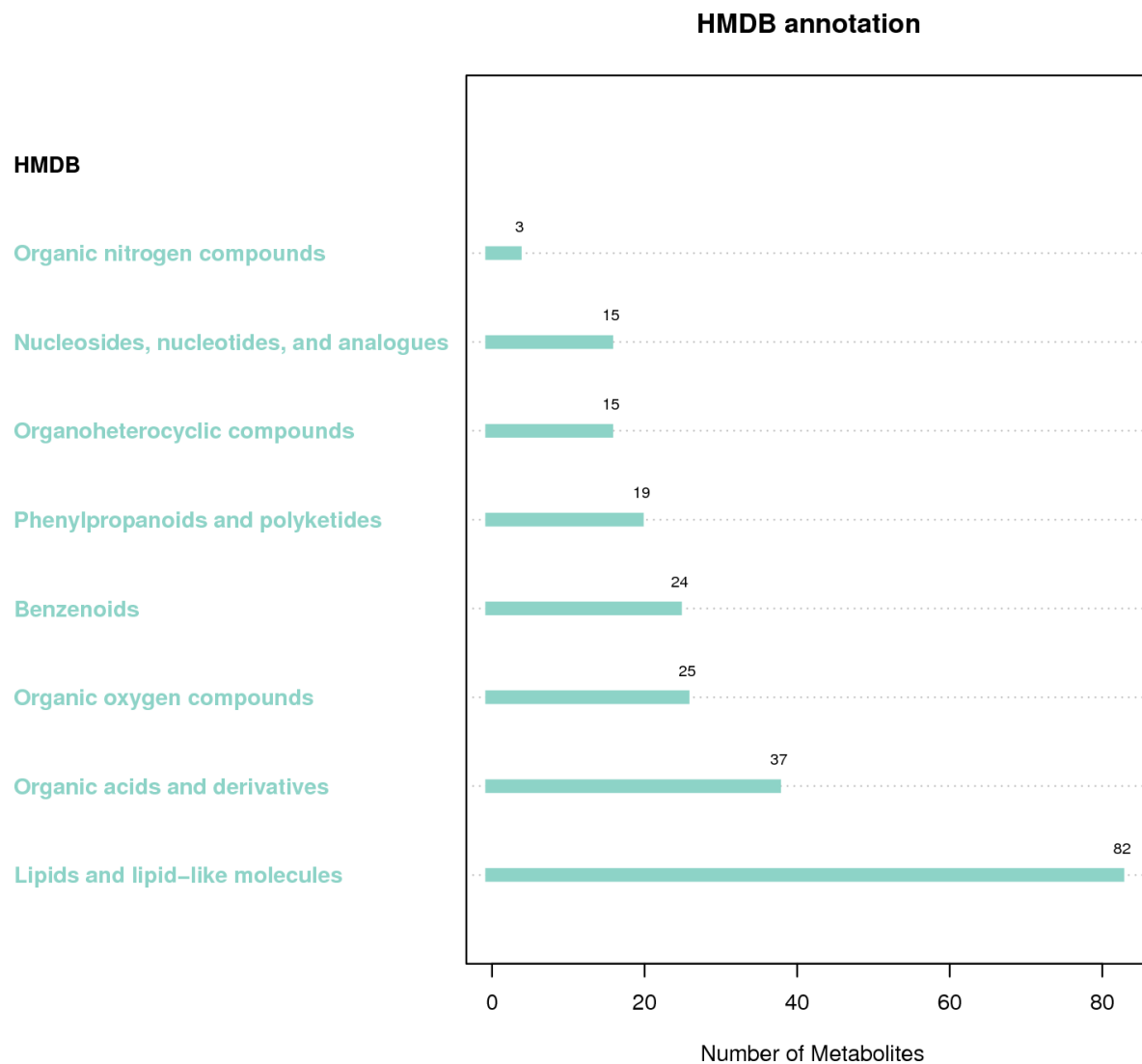

**Figure S3.** HMDB annotation results.

Supplement: Supplementary file 1 [file foods-11-03318-s001.zip › Figure S3.pdf]
